# Licensing Adaptive Immunity by NOD-Like Receptors

**DOI:** 10.3389/fimmu.2013.00486

**Published:** 2013-12-27

**Authors:** Dong Liu, Anne Marie Rhebergen, Stephanie C. Eisenbarth

**Affiliations:** ^1^Department of Laboratory Medicine, Yale University School of Medicine, New Haven, CT, USA; ^2^Department of Immunobiology, Yale University School of Medicine, New Haven, CT, USA; ^3^Department of Internal Medicine, Yale University School of Medicine, New Haven, CT, USA

**Keywords:** NLR, dendritic cell, NLRP3 inflammasome, NLRP10, vaccine response, autoimmunity, Th2 response, asthma

## Abstract

The innate immune system is composed of a diverse set of host defense molecules, physical barriers, and specialized leukocytes and is the primary form of immune defense against environmental insults. Another crucial role of innate immunity is to shape the long-lived adaptive immune response mediated by T and B lymphocytes. The activation of pattern recognition receptors (PRRs) from the Toll-like receptor family is now a classic example of innate immune molecules influencing adaptive immunity, resulting in effective antigen presentation to naïve T cells. More recent work suggests that the activation of another family of PRRs, the NOD-like receptors (NLRs), induces a different set of innate immune responses and accordingly, drives different aspects of adaptive immunity. Yet how this unusually diverse family of molecules (some without canonical PRR function) regulates immunity remains incompletely understood. In this review, we discuss the evidence for and against NLR activity orchestrating adaptive immune responses during infectious as well as non-infectious challenges.

## Introduction

When pathogens or injuries threaten the body, two different branches of the immune system work together to restore homeostasis: the innate and adaptive immune systems. They monitor the tissues of vertebrates and use different tactics to recognize and overcome threats. These two branches are inherently different in the sensors and mechanisms employed in order to provide either immediate protection with broad specificity (innate immunity) or delayed and prolonged protection with exquisite specificity (adaptive immunity). Further, the two branches must effectively coordinate a response in order to prevent excessive or inappropriately targeted inflammation. As Charles Janeway wrote in 1992, “the immune system evolved to discriminate infectious non-self from non-infectious self” (1). We will use this paradigm to develop a broadened classification system of five adaptive immune response types and then review how nucleotide-binding oligomerization domain-containing receptors (NOD-like receptors or NLRs) potentially regulate the response to each. These five categories are based on the origin of the target antigen affected by the adaptive immune response: (1) foreign pathogenic targets (i.e., antimicrobial immunity); (2) foreign non-pathogenic targets (i.e., allergies); (3) self targets (i.e., autoimmunity); (4) altered self targets (i.e., tumor immunity); and (5) foreign self targets (i.e., commensal homeostasis). The role of NLRs in each category is likely different, as the initiation of immunity as well as the outcome for the host are different (e.g., beneficial protection to pathogens versus loss of tolerance resulting in self-destruction).

### Pattern recognition receptors in innate and adaptive immunity

The innate immune system consists of barriers, networks of soluble mediators, and myeloid-derived executioner (effector) cells including macrophages, dendritic cells (DCs), and granulocytes. It utilizes evolutionary conserved receptors to survey the extracellular and intracellular environment for pathogenic elements and injury. These molecules include pattern recognition receptors (PRRs) and as the name suggests, they are able to recognize a variety of common molecular motifs called “pathogen/microbe associated molecular patterns” (PAMPs/MAMPs) derived from microbes, and “damage associated molecular patterns” (DAMPs) derived from mislocalized or damaged host molecules during states of cell stress (2). PRRs exist in transmembrane, secreted, and cytosolic forms. Toll-like receptors (TLRs) are located on the cell surface and in endosomal compartments where they can recognize extracellular or phagocytosed pathogens (3). C-type lectin receptors (CLRs) can be found in both membrane-bound and secreted forms, and bind carbohydrate-based PAMPs and DAMPs (4). RIG-I-like receptors (RLRs), AIM2-like receptors (ALRs), and other nucleic acid sensing PRRs along with NLRs are located exclusively in the cytosol and nucleus, where they detect pathogens or processes that breach the cell membrane (2, 5–7). NLRs respond to a wide variety of PAMPs and DAMPs as well as intracellular and extracellular signals generated by other arms of the innate and adaptive immune system. Following PRR activation, various signaling cascades are induced that initiate or shape the appropriate inflammatory response and, mostly through the action of DCs, activate T and B lymphocytes of the adaptive branch of the immune system. As the highly specific antigen receptors on lymphocytes can sense an almost infinite diversity of antigens, a crucial distinction between an “appropriate” and an “inappropriate” target for the adaptive immune response is made during this step.

### NLR family architecture and functions

**Figure 1:**
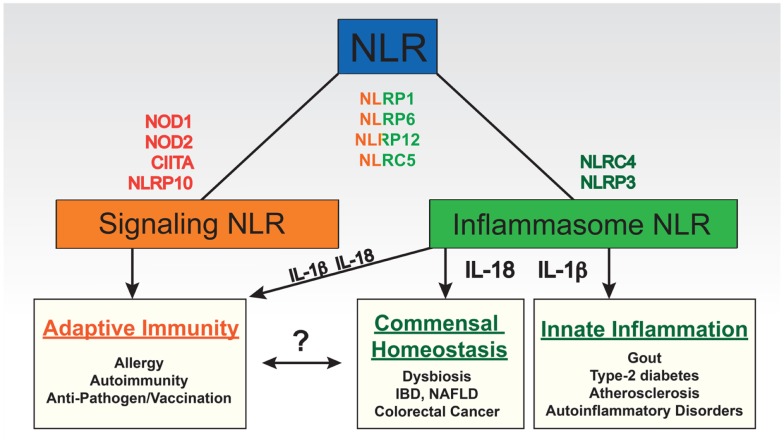
**Functional categories of NLRs based on their roles in shaping immune responses**. Some NLRs have been found to play primary roles in regulating particular pathways (e.g., transcription) or signaling cascades (“signaling NLRs”) such as NOD1, NOD2, NLRP10 (putative signaling NLR), and CIITA. Others regulate the formation of inflammasomes (“inflammasome NLRs”) such as NLRP3 and NLRC4. Some do both such as NLRP1, NLRC5, NLRP6, and NLRP12. The immune consequences of these broad categories can be quite different. Although IL-1β and IL-18 derived from inflammasome activity can regulate particular aspects of adaptive immunity, direct roles of each of these cytokines in promoting non-lymphocyte based inflammatory reactions or commensal flora in the gut have also been identified. The latter has been implicated in shaping the adaptive immune system and therefore we linked these two categories; however, no direct role has yet been identified for an NLR in this link. On the other hand, NLRs involved in signaling cascades that in particular effect dendritic cell function as antigen presenting cells have a more obvious direct effect on adaptive immunity. IBD, inflammatory bowel disease; NAFLD, non-alcoholic fatty liver disease.

The array of cellular responses regulated by NLRs is striking and includes transcription (e.g., of MHC molecules), enzymatic activity (e.g., of caspases), and positive and negative regulation of intracellular signaling cascades (e.g., NF-κB pathway) (Figure 1). This latter category of signaling NLRs primarily modulates pathways relevant to the innate immune response or its regulation of adaptive immunity. Signaling through NOD1 and NOD2, after sensing various bacterial peptidoglycan fragments, results in the activation of pro-inflammatory NF-κB and MAPK pathways, and the induction of autophagy (8–11). Extensive work on the biochemistry, ligands, and role of NOD1 and NOD2 in the innate and adaptive immune response has been done and is the topic of an accompanying review by Boyle et al. (12). NLRP10 and NLRP12 are required for the migration of antigen presenting cells (APCs) and thus have a role in defining the onset of adaptive immunity as will be discussed further in subsequent sections (13, 14). The NLRs CIITA and NLRC5 are induced by cytokines and act as transcriptional activators of MHC molecules, thereby potently regulating adaptive immunity. CIITA controls the transcription of MHC class II molecules and related proteins necessary for the presentation of antigen to CD4^+^ T cells; NLRC5 does the same for MHC class I molecules and related proteins (15, 16) although it appears to have a broader function in pathogen sensing as well (17–19). In addition, several NLRs appear to have functions unrelated to the recognition of pathogens or damage, such as tissue homeostasis and embryonic development; however, further research is needed to unravel their exact roles (20).

Some NLRs perform more than one of these functions and for a majority of NLRs no known function has yet been identified. Therefore NLRs are classified by their structure, which consists of three distinct parts: a central nucleotide-binding domain (NBD), a carboxy-terminal leucine-rich repeats (LRR), and an amino-terminal effector domain. The effector domain instigates activity and divides NLRs into four subfamilies: the NLRA family, with an acidic domain, the NLRB family, with a baculoviral inhibitory repeat (BIR) domain, the NLRC family, containing a caspase activation and recruitment domain (CARD) and the NLRP family, which has a pyrin domain. Only NLRP1 and NLRP10 do not have this basic structure (21, 22). The only domain common to all NLRs is the central NBD, which has ATPase activity and is thought to induce oligomerization of the NLR proteins following activation (5).

### NLR activation

The classic paradigm for the function of a pattern recognition receptor was established through the study of TLRs. TLRs sense a foreign motif (e.g., a PAMP) on a pathogen through receptor ligation of the LRR domain, resulting in the induction of an inflammatory response and enhanced antigen presentation to lymphocytes [reviewed in Ref. (6)]; however, this paradigm does not fit the NLRs. Except for a few members like NOD1, NOD2, and NAIP5 (23–27), there is limited direct evidence that the LRR actually recognizes their respective agonists (28). It has been difficult to precisely define how NLRs bind ligands, activate, and oligomerize, although from electron microscopy data, a wheel-like structure analogous to many of the other oligomerized STAND (signal transduction ATPases with numerous domains) proteins appears to form (29–31). A recent crystal structure of part of NLRC4 in the inactive state suggests that the activity of this NLR is indeed regulated by the LRR domain along with the adjacent helical domain HD2 (32). Yet it had been previously demonstrated that instead of directly interacting with its ligands, NLRC4 senses PAMPs with the help of adaptor NLRs NAIP2 and NAIP5, which associate with PrgJ and flagellin of flagellated bacteria, respectively, and control the oligomerization of NLRC4 after ligand binding (25, 33).

Despite the many known stimuli, including insoluble crystals, bacterial toxins, and extracellular ATP, the mechanism by which NLRP3 is activated is only recently becoming clear. Because of the diversity of these ligands, it is likely that they activate NLRP3 through a shared mechanism involving desequestration of a host-derived trigger (34, 35). Sensing of increased cytosolic reactive oxygen species (ROS), intracellular calcium fluxes (36–38), potassium efflux (39–42), protein kinase (PKR) binding (43), or the release of contents from phagolysosomes have all been proposed as mechanisms. Numerous recent studies have honed in on mitochondrial-derived triggers regulating the cellular processes listed above or release of NLRP3 ligands from mitochondria including ATP, DNA, ROS, or mitochondria-associated adaptor molecule (MAVS) (38, 44–51). Given the importance of the mitochondria in regulating cell death, the potential triggering of NLPR3 activity by mitochondrial-derived signals suggests an interesting and central role for this organelle in integrating numerous cellular insults into a set of cell fate outcomes, some of which might be NLR-dependent (52). Biochemical identification of NLRP3 and other NLR-specific ligands, whether PAMPS, DAMPS, or more traditional signaling pathway molecules, will greatly facilitate our understanding of how NLRs shape both innate and adaptive immunity.

### Inflammasome complexes

After recognition of a PAMP or DAMP, a number of NLRs (NLRP1, NLRP3, NLRP6, NLRP7, NLRP12, NLRC4, NLRC5, NAIP2, NAIP5) form multi-protein complexes called inflammasomes (19, 33, 53–56). The signature events of a functional inflammasome are the activation of caspase-1 and subsequent cleavage of the pro-inflammatory cytokines IL-1β and IL-18 into their bioactive forms. Inflammasomes are thought to consist of multiple copies of an NLR that, after ligand sensing, recruit the protease pro-caspase-1. In most inflammasomes, these proteins are oligomerized by an adaptor protein called ASC (apoptosis-associated speck-like protein containing a CARD). ASC consists of both a pyrin domain and a CARD domain, which enable it to interact with the pyrin domain of the NLR and the CARD domain of pro-caspase-1. NLRC4 and mouse NLRP1b do not need ASC to form an inflammasome, but when ASC is recruited, the production of cytokines following NLRC4 signaling is much more efficient (57–59). However, since no NLR inflammasome structure has yet been solved (30, 60), there is still debate on the exact composition of inflammasomes. A “non-canonical” pathway resulting in inflammasome function has recently been described, in which caspase-11 activation by cytosolic Gram-negative bacteria such as *Escherichia coli* and *Citrobacter rodentium* enhances caspase-1 inflammasome activity or instigates cell death (61–63).

## How NLRs Might Shape the Five Categories of Adaptive Immune Responses

### Dendritic cells in innate and adaptive immunity

The numerous pathogens we encounter everyday do not normally cause disease because most are quickly eliminated by our innate immune system. Under certain circumstances, some pathogens can evade our first line of defense and our adaptive immune system must use alternative tactics to combat the invading pathogen. APCs are critical for initiating this adaptive immune response by processing antigen and presenting it to naive T cells. Among APCs, DCs are thought to be the most potent as they temporally express requisite T cell co-stimulatory molecules, are readily motile and are widely distributed throughout the body forming a remarkable network of sentinel cells (64, 65). Under the steady state, DCs patrol the body seeking out evidence of invasion or malfunction. They express a variety of PRRs, including TLRs, NLRs, RLRs, and CLRs; PRRs are one major pathway DCs use to recognize either foreign invaders (containing PRR-stimulating PAMPs) or self molecules that have become altered (e.g., dying or transformed cells containing DAMPs). PRR activation dramatically impacts DC function by altering antigen presentation, phagocytic and macropinocytic capacity, and migratory properties. This allows DCs in peripheral tissues to transmit information about infection or tissue damage to the distal secondary lymphoid organs where naïve T-cells await stimulation.

**Figure 2:**
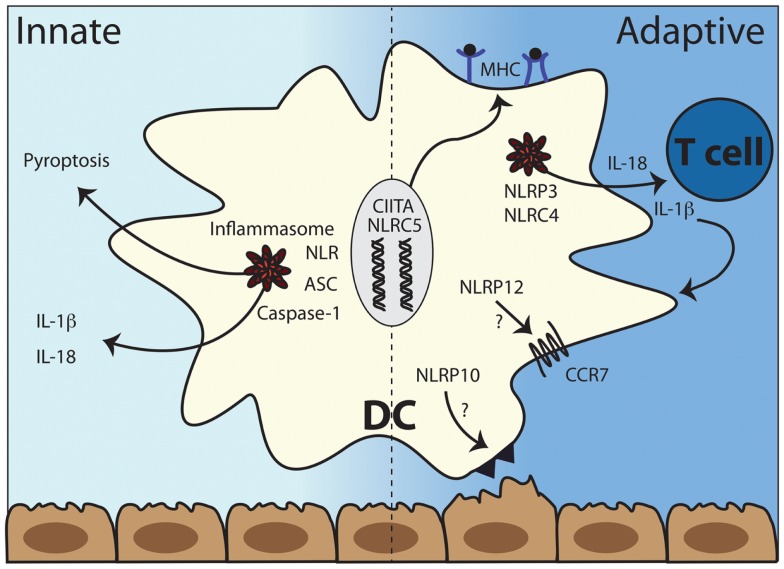
**The multiple roles of NLRs in dendritic cell function**. Left panel: the recognition of intracytoplasmic triggers leads to the formation of inflammasomes and subsequent cleavage and release of the pro-inflammatory cytokines IL-1β and IL-18 and a specific kind of cell death called pyroptosis. Right panel: antigen presentation depends critically on CIITA and NLRC5 while inflammasome activation results in IL-1β and IL-18 production, which can have both autocrine effects on DC function as well as shape the differentiating T cell cytokine profile. NLRP10 and NLRP12 both are involved in DC migration from peripheral tissues to draining lymph nodes although potentially via different mechanisms. DC, dendritic cells; ASC, apoptosis-associated speck-like protein containing a CARD; NLR, NOD-like receptor; CCR7, C–C chemokine receptor type 7, MHC, major histocompatibility complex (class I left and class II right).

The role of TLRs in shaping adaptive immunity via modulating DC function is well-studied, while the role of NLRs in adaptive immunity is less well understood. Recent studies suggest that the NLRs NOD1 and NOD2 might influence T cell differentiation via enhancing cytokine production from DCs in synergy with TLRs (66, 67). Work from our group and others demonstrated that particular NLRs can regulate DC migration during inflammation and infection (13, 14, 68). Antigen presentation depends critically on CIITA and NLRC5 while inflammasome activation results in IL-1β and IL-18 production, which can have both autocrine effects on DC maturation as well as shape the differentiating T cell cytokine profile (Figure 2). Despite these few examples, most of the work on NLRs has not clearly elucidated how DCs are affected by NLR activity. We will discuss those studies that have addressed the role of NLRs in shaping each of the five categories of adaptive immunity defined above and highlight findings regarding DCs when known.

## Category 1: Adaptive Immunity to Foreign Pathogenic Targets

Unlike the well-studied TLRs, which detect extracellular or endosomal PAMPs, the NLRs respond to pathogens that have reached the cytosol or the accompanying cell damage caused by breaching the cell membrane. Accordingly, NLRs can respond to particular pathogens that might evade detection by TLRs or other PRRs. For those NLRs that have been studied, much of the work regarding immune consequences of NLR activity has either used *in vitro* systems or evaluated the acute innate immune responses *in vivo*. Therefore knowledge regarding how NLRs shape adaptive immunity during infection is largely incomplete. In this section we will discuss only those studies that addressed regulation of adaptive immune responses to bacterial, viral, parasite, or fungal pathogens.

### Bacteria

Nucleotide-binding oligomerization domain-containing receptors are well suited to detect live cytosolic bacteria via PAMP recognition such as flagellar proteins or the cellular effects invading bacteria induce such as pore formation by toxins or secretion systems. The exotoxin pneumolysin (PLY) from *Streptococcus pneumoniae* is an important virulence factor responsible for forming pores in cell membranes (69). It was previously shown that PLY was crucial for caspase-1 activation and subsequent IL-1β and IL-18 production during pneumococcal infections (70, 71). In line with this, a recent study demonstrated that PLY activation of the NLRP3 inflammasome synergized with TLR agonists on *S. pneumoniae* to enhance secretion of IL-1β, IL-1α, and TNF-α by DCs and promote IL-17A and IFN-γ production by splenocytes (72, 73). Accordingly, NLRP3-deficient mice were more susceptible to pneumococcal pneumonia, suggesting that the inflammasome and its components contribute to the protective immune response to this bacterium.

A particularly crucial NLR poised to detect intracellular pathogens and orchestrate the subsequent adaptive immune response is NLRC5. Although NLRC5 has been reported to have a variety of functions in the innate immune response, its role in transcriptional regulation of MHC class I gene expression is essential for driving CD8^+^ T cell priming to intracellular bacteria such as *Listeria monocytogenes* (18). NLRC5 expression is enhanced by PAMP as well as IFN-γ stimulation of hematopoietic cells and it can move from the cytosol into the nucleus where it binds and transactivates mouse and human class I genes as well as associated genes such as β2 microglobulin and TAP1 (74, 75). Therefore NLRC5 has a fundamental role in antigen presentation. The role of NLRs is less clear in another intracellular bacterial infection, *Mycobacterium tuberculosis (Mtb)*. *Mtb* readily infects macrophages *in vitro*, which subsequently secrete IL-1β and IL-18 in a NLRP3 inflammasome-dependent manner (76–79). As signaling through the IL-1 receptor and MyD88 are required for protection during *Mtb* infection, inflammasome regulation of protective immunity seemed likely. Using heat-killed *Mtb* as part of the potent adjuvant complete Freund’s adjuvant (CFA), Shenderov and colleagues indeed demonstrated a profound impairment of Th17 differentiation in ASC- and caspase-1-deficient mice secondary to reduced IL-1β stimulation of T cells (80). Yet *in vivo* with a live infection, IL-1β levels in the lung are unaffected by loss of ASC or caspase-1 and bacterial burden and mortality in inflammasome-deficient mice are not significantly different from WT mice (76, 77, 79). Although IL-1β processing and secretion are, under most circumstances, inflammasome dependent, IL-1α is often secreted in an active form in an inflammasome-independent fashion (81). As both cytokines activate IL-1R1 and either cytokine alone is sufficient for protection during *Mtb* infection, it is perhaps not surprising that NLRP3 and the associated inflammasome components are dispensable for protective immunity (82).

Although numerous studies on NLRC4 have described its role in the innate immune response to flagellated bacteria, only a few investigations evaluated the role of NLRC4 in generating adaptive immunity to bacteria. Flagella from *Salmonella, Yersinia*, and *Pseudomonas* activate the NLRC4 inflammasome in splenic CD8α^+^ DCs to release IL-1β and IL-18; IL-18 can stimulate IFN-γ production from non-cognate memory CD8^+^ T cells in the spleen thereby enhancing host resistance to bacterial infection (83). Caspase-1 regulated IL-1β and IL-18 production from DCs was also crucial for both Th1 and Th17-dependent protective immunity during *Helicobacter pylori* infection or even following vaccination, although the relevant NLR involved remains unknown (84). Caspase-1-, ASC-, and IL-18-deficient mice are all more susceptible to infection with the intracellular bacterium *Anaplasma phagocytophilum* as a result of inadequate Th1 activity. Partially via the inflammasome-forming activity of NLRC4, enhanced IL-18 secretion (presumably by APCs in the spleen) promotes Th1 differentiation and IFN-γ production – a crucial cytokine in regulating *A. phagocytophilum* bacterial loads (85). In fact one evasion mechanism used by the pathogen *Salmonella* is to downregulate NLRC4 expression to prevent the inflammasome response and thereby promote bacterial persistence and dissemination (86). Therefore NLRC4 and its adaptors are well poised to detect intracellular bacteria and mount an important inflammasome-dependent IL-18 driven adaptive immune response.

### Viruses

Toll-like receptor- and RIG-I-like receptor-mediated recognition of viral infections results in the production of a number of viral resistance pathways and in particular the secretion of type I IFNs; in contrast, NLRs do not directly regulate IFNα or IFNβ and in fact inflammasome-driven pathways can be inhibited by these cytokines. Most NLRs do not directly recognize viral motifs but rather respond to cell disruption induced during viral infection with inflammasome-dependent cytokine secretion (87–89). The most extensively studied virus that activates inflammasome pathways is the respiratory pathogen influenza. Much like the pore forming bacteria described above, influenza can activate the NLRP3 inflammasome via insertion of a proton-selective ion channel, which it uses to change ionic gradients in intracellular compartments during viral entry and propagation, but also alerts NLRP3 to presence of the virus (90).

It is well known that IL-1 cytokines are crucial to the Th1 and CD8^+^ T cell adaptive immune response to influenza; accordingly, ASC and caspase-1 are also needed for protective adaptive immunity (91, 92). A recent paper showed that DCs from elderly mice exhibited decreased expression of ASC, NLRP3, and caspase-1 compared with DCs from infected young mice and the concomitant blunted IL-1β response resulted in enhanced morbidity and mortality in influenza-infected elderly mice (93). A similar study demonstrated that TLR3 or NLRP3 stimulating adjuvants enhanced the efficacy of influenza virus-like particle vaccines in aged mice (94). These studies suggest that suppressed NLRP3 inflammasome activity during aging impairs protective adaptive immunity to influenza. Yet the course of influenza infection in different studies using NLRP3-deficient mice widely varied with some studies showing enhanced influenza susceptibility and others showing no difference (92, 95, 96). It was suggested that these opposing results might be due to different doses of influenza virus inoculation or the different viral strains used in these studies (92), but an alternative explanation might come from recent data on the role of IL-1 cytokines in regulating DC migration during influenza infection. Pang et al. found that IL-1 signaling in pulmonary DCs was required for proper DC migration to lung draining lymph nodes and subsequent activation of influenza specific T cells; however, DC intrinsic activation of NLRP3 was not needed for antigen presentation or T cell priming (97). Therefore, much like the pathways described for *Mtb*, if inflammasome-independent IL-1α can be generated during infection, adaptive immunity should be minimally impacted in the absence of NLRP3, ASC, or caspase-1.

West Nile virus (WNV) is an emerging flavivirus capable of infecting the central nervous system (CNS) and mediating neuronal cell death. A recent study demonstrated that acute WNV infection induced IL-1β production *in vivo*, and that IL-1-R-, caspase-1-, and NLRP3-deficient mice exhibited increased susceptibility to WNV (98). This outcome was associated with increased accumulation of virus within the CNS and reduced anti-viral activity of effector CD8^+^ T cells. This study indicates that IL-1β signaling and the NLRP3 inflammasome are important for host control of virus replication in neurons and WNV-induced pathology in the CNS.

### Parasites

Although the NLRP3 inflammasome is activated during many parasitic infections (e.g., *Leishmania major*) or by byproducts of parasitic infections (e.g., malarial hemozoin) and its effect on the acute innate immune response is well documented, the role of NLRP3 or other NLRs in the adaptive immune response to parasites has not been extensively studied (99, 100). The role of the NLRP3 inflammasome in shaping the T cell response to *Schistosoma mansoni* has been studied (101); the authors found that both ASC- and NLRP3-deficient mice failed to upregulate IL-1β in the liver and all parasite-specific helper T cell responses (Th1, Th2, and Th17) were reduced after infection compared to wild type controls. Surprisingly, these impaired T cell responses were correlated with smaller liver granulomas and attenuated immunopathology in the inflammasome-deficient mice, suggesting enhanced adaptive immunity to *Schistosoma* by NLR activation is not necessarily beneficial to the host.

### Fungi

It has been reported that *Candida albicans* hyphae activate NLRP3 (102, 103) and NLRP3-deficient mice succumb to both disseminated Candidiasis (102, 104) and mucosal Candidiasis (103), suggesting that the inflammasome is crucial for anti-fungal host defense. Caspase-1- and ASC-deficient mice indeed have impaired Th1 and Th17 responses during *Candida albicans*, leading to increased fungal outgrowth and reduced survival (105). Further, exogenous IL-18 was able to restore Th1 immunity to *Candida* in caspase-1-deficient mice (106), indicating that inflammasome-derived cytokines direct protective adaptive immune responses during invasive fungal infection.

NLRP10 has a critical role in DC localization *in vivo*; in its absence, impaired DC migration results in a profound helper T cell priming defect in a number of immunization models (14). In line with this, NLRP10-deficient mice have increased susceptibility to disseminated Candidiasis, as indicated by decreased survival and increased fungal burdens, secondary to impaired induction of *Candida*-specific Th1 and Th17 responses (68). In fact, adoptive transfer of *Candida*-specific primed T cells from WT mice rescues NLRP10-deficient mice infected with *Candida*. Therefore NLRP10, by regulating the movement of DCs presumably in the spleen in this case, can dramatically regulate pathogen-specific adaptive immunity. It still remains to be determined how NLRP10 activity is triggered during *Candida* infection and the molecular function of NLRP10 that regulates DC migration.

### Clinically approved vaccine adjuvants

The development of vaccines against infectious pathogens has been and continues to be one of the most important medical interventions in global health. Many of our current vaccines do not induce adequate immunity unless co-administered with an adjuvant, which helps initiate adaptive immunity by stimulating the innate immune system. Although many pathogen-derived adjuvants used in animal models (e.g., CFA containing heat-killed mycobacteria or immunostimulatory oligodeoxynucleotides containing CpG motifs) have the capacity to stimulate various TLRs and NLRs, almost all clinically approved adjuvants for human use in fact do not contain any pathogen-derived motifs. Instead the most commonly employed adjuvants in human vaccines are aluminum hydroxide, which is a crystalline mixture and MF59, which is an oil-in-water emulsion. We and a number of other groups hypothesized that these adjuvants might trigger a DAMP-dependent rather than PAMP-dependent innate immune response thereby accounting for their immunostimulatory properties. Indeed alum is a potent activator, like many other insoluble crystals, of the NLRP3 inflammasome (107–111); however, whether triggering this innate immune pathway subsequently instructs the adaptive immune response during vaccination remains unclear (112). Different groups using the same mice with similar models have observed opposing phenotypes, possibly suggesting that multiple factors influence whether NLRP3 is relevant to the adjuvant function of alum. Some studies, including our own, have suggested that induction of adaptive immunity following alum-based vaccination requires a functional NRLP3 inflammasome via maturation of DCs (107, 109, 110); however, other groups found that NLRP3-deficient mice had intact T and B cell responses following vaccination with alum and antigen (108, 111, 113). Further, numerous other pathways downstream of alum immunization have now been proposed to be required for its adjuvant function, including DNA released during cell death, lysosomal permeabilization resulting in cathepsin B and S release, extracellular ATP, and uric acid release (114–117). Surprisingly, all of the above stimuli have also been directly implicated in NLRP3 inflammasome activation, yet in all cases NLRP3 was not required for successful immunization, leaving us without consensus on the mechanism by which alum regulates adaptive immunity. As alum has proven to be a great adjuvant for almost 100 years in that it works in most people, it is likely that it has multiple routes to induce immunity. In isolated systems used by different groups using murine models we have uncovered some, but perhaps not all, of those mechanisms.

A similar story has emerged for the oil-in-water adjuvant MF59 (118, 119). Ellebedy et al. showed that although NLRP3 is dispensable for the adjuvant effect of MF59, ASC is crucial for the induction of antigen-specific IgG following vaccination with H5N1 in combination with MF59 (118). Indeed, activation of both germinal center B cells and CD4^+^ T cells are significantly reduced in ASC-deficient mice, but not in NLRP3- or caspase-1-deficient mice. As it has been shown that DCs take up both antigen and MF59 and transport them to draining lymph nodes (120, 121), the markedly reduced secretion of inflammatory cytokines by ASC-deficient DCs upon stimulation with MF59 may be responsible for impaired T and B cell responses (118). Consistent with these findings, Seubert et al. also demonstrated that NLRP3 is not required for induction of adaptive immune responses to *Neisseria meningitides* with three particulate adjuvants: alum, MF59, and CFA (119).

Apart from traditional adjuvants, nanoparticles have been extensively studied as vehicles to co-deliver antigens and adjuvants for effective vaccination (122). Using a biocompatible polyester, poly(lactic-co-glycolic acid) (PLGA), loaded with antigen Demento et al. demonstrated that LPS-modified particles are preferentially internalized by DCs compared to uncoated nanoparticles and elicit NLRP3 inflammasome activity (123). Further, inhibition of endocytosis and lysosomal destabilization diminished inflammasome activity, suggesting that the rupture of lysosomal compartments by nanoparticles is a crucial trigger for inflammasome activation (123, 124). A more recent study found that porous silicon nanoparticles enhance phagocytosis and subsequent activation of DCs as well as IL-1β production (125). Administration of these nanoparticles also enhances DC migration to draining lymph nodes and T cell priming. However, adaptive immunity was either not evaluated in these studies or only partially affected in the absence of NLRP3, again leaving us with a vaccine formulation that can activate the inflammasome but might not absolutely require its activity for the initiation of adaptive immunity.

Nucleotide-binding oligomerization domain-containing receptors not only perform a major role in innate immune responses, but also have defined roles in generating effective adaptive immune responses against various infections. However, a number of important questions remain unanswered, including the mechanism by which NLRs detect molecules of microbial origin as well as how NLRs cooperate with other PRRs to mount an effective immune response against pathogens. It is possible that some NLRs serve as direct receptors of PAMPs, while others instead detect the perturbation of the host microenvironment by pathogens (42). Undoubtedly, understanding how NLRs recognize microbial products and initiate subsequent adaptive immune responses will provide new insights into vaccine design and vaccination strategies against infectious diseases.

## Category 2: Adaptive Immunity to Foreign Non-Pathogenic Targets

The diversity of molecules that can act as allergens is immense with the only unifying theme being the allergic type 2 immune response induced; in most cases this immune response is driven by Th2 lymphocytes [for review, see (126)]. Allergens are foreign, non-self molecules, and as such could trigger innate immune responses. Yet as most [but not all (126)] of these molecules are considered inherently non-pathogenic, it is unclear if they trigger classic PRR pathways. Many allergens indeed have enzymatic activity or are pathogen-derived (e.g., helminths) or can mimic PAMPs (e.g., Derp 2) and this might be how the innate immune system is tricked into initiating adaptive immunity (127, 128). Are NLRs involved in sensing allergens as pathogenic and determining the subsequent maladaptive immune response? Given that some allergens can directly induce tissue damage along with previous implications of IL-1 and IL-33 in Th2-mediated inflammatory responses, the prediction that NLRs play a role in the development of allergic diseases seems obvious. However, the evidence in favor of this argument is limited (129).

IL-33 is a prime candidate in the development of allergy as it clearly promotes Th2 (as well as ILC2) responses (130–133). Early work in the inflammasome field suggested that IL-33 was cleaved by caspase-1 and so this cytokine was grouped with the other inflammasome-regulated cytokines IL-1β and IL-18 and further postulated to be one mechanism by which inflammasome activity could regulate Th2 development (107). However, more recent work indicates that active IL-33 is in fact released from necrotic cells and functions more like a DAMP; if IL-33 is instead cleaved by caspase-1 or by caspase-3 during apoptosis it becomes inactive and fails to bind its receptor T1/ST2 (134–136). Therefore, the data supporting a role for IL-33 in the development of Th2-driven allergic inflammation indicates that this cytokine can function like a DAMP and shape the immune response but does not implicate a role for inflammasome activity in such a process.

### Lung

Similar to the diversity of antigens that can serve as allergens, there is a spectrum in the pathophysiology of the major allergic lung disease, asthma. The classic form is triggered by inhaled allergens that trigger degranulation of mast cells and basophils in the lung in an IgE-dependent manner and results in airway constriction and mucus production along with tissue inflammation dominated by activated eosinophils. Although the IL-1 gene cluster on chromosome 2 and promoter polymorphisms in IL-18 have been weakly associated with susceptibility to asthma (137, 138), the mechanistic link between IL-1, inflammasome activity and Th2 induction is uncertain partly due to the range of allergic airway disease models employed. As many murine allergy models involve intraperitoneal administration of antigen with the potent adjuvant aluminum hydroxide, the role of NLRs during the T cell sensitization phase to allergens in lung draining lymph nodes is difficult to determine (139); indeed intraperitoneal injection models have demonstrated a range of effects in IL-1- or IL-1 receptor-deficient or inflammasome-deficient mice (113, 140, 141). In contrast, when inhalational allergens are administered with nitrogen dioxide, urban particulate matter, or with presumed low-level PAMP contamination, a more significant role for IL-1 pathways and inflammasome molecules has been observed in some (142–145) but not all allergic airway disease models (113, 141). One caveat to these latter systems however, is that they can have a significant Th17-driven inflammatory response and as some studies have demonstrated a significant role for inflammasome-derived IL-1β on Th17 but not Th2 induction, the observed differences in inflammation might be due to loss of the Th17 component (143, 146). Nevertheless, inflammasome activity likely via IL-1 secretion appears to modulate particular aspects of adaptive immunity to inhaled triggers.

### Gastrointestinal

The disease corollary of this type of allergic adaptive immune response in the gastrointestinal system is food allergies. Outside of one study on subjects with a specific clinical subset of food allergies demonstrating an association with *Nlrp3* polymorphisms (147), there has been no link yet established between NLRs and the development of adaptive immunity to food antigens.

### Skin

The other primary organ affected by allergic adaptive immunity is the skin. Disorders such as atopic dermatitis (in some cases referred to as eczema) and allergic contact dermatitis (included under contact hypersensitivity) are manifestations of an often Th1/Th2 mixed adaptive immune response to unclear targets, but can include prototypical allergens such as house dust mite antigens or small molecule contact allergens. Accordingly, the inflammasome-dependent cytokines IL-1β and IL-18 have been implicated in promoting aspects of both a Th2 (elevated IgE, mast cell, and basophil degranulation with overproduction of type 2 cytokines) and a Th2- and IgE-independent innate inflammatory response (basophil and mast cell degranulation) (148, 149). IL-18, traditionally thought of as a Th1-promoting cytokine, has been implicated in the development of atopic dermatitis; transgenic mice expressing either human caspase-1 or IL-18 in keratinocytes develop spontaneous atopic dermatitis-like lesions with elevated mast cell activity (148). House dust mite cysteine proteases can directly activate the NLRP3 inflammasome in keratinocytes, which has been one proposed mechanism for induction of caspase-1 activity (150); however, mast cell chymase can also directly cleave pro-IL-18 independently of caspase-1 (151). Further work is needed to clarify if particular NLRs are indeed needed to direct the adaptive immune response to skin antigens in atopic dermatitis, but clearly pathways downstream of particular NLRs appear relevant.

Contact hypersensitivity is triggered by contact allergens (haptens, most often small lipophilic molecules that bind to self proteins) that induce a Th1/CD8+ T cell driven neutrophilic inflammation. An important role for IL-1β in the development of contact hypersensitivity has been known for many years (152) and a role for inflammasome-forming NLRs seems likely, especially given the expression of many NLRs and associated inflammasome components in the skin (153). Multiple studies have suggested that IL-1β directly enhances Langerhans cell activity in priming T cells to contact allergens (154, 155). However, subsequent work on this unique cell has indicated that it is primarily derived from embryonic fetal liver monocytes and locally maintained in contrast with classical DCs; further, Langerhans cells migrate significantly slower (e.g., on the order of 3–4 days) to draining LNs than typical DCs (156, 157). Recent studies instead suggested a regulatory role of Langerhans cells to skin-derived antigens (157). Therefore, the exact role of Langerhans cells in initiating adaptive immunity to contact sensitizers is not clear. Yet separate studies using caspase-1, ASC, or NLRP3-deficient mice all support a requirement for inflammasome activity in the generation of immunity to skin sensitizers (153, 155, 158, 159). This implies that inflammasome-regulated IL-1β might target cells other than Langerhans cells during sensitization in the skin such as dermal DCs; alternatively, inflammasome-dependent signals beyond IL-1β might directly modulate the lymphocyte response to contact sensitizers.

Beyond regulating inflammasome-induced cytokine secretion, NLRs can play a more direct role in regulating sensitization of T cells via affecting DC maturity and migration. Recently two NLRs were described to regulate the movement of DCs in an inflammasome-independent manner and gene knockouts of each had dramatic but specialized effects on allergic models *in vivo*. Arthur et al. demonstrated that hapten-induced contact hypersensitivity was defective in the absence of inflammasome-forming NLRP12 potentially via regulation of DC and neutrophil motility but independent of inflammasome activity (13). However, subsequent work from the same group indicated that NLRP12-deficient mice had no defect in T cell sensitization to antigens inhaled or administered intraperitoneally in two different allergic airway disease models (141), suggesting that NLRP12-dependent DC migration might be specific to skin DCs. NLRP10 was described by a number of groups including our own to regulate DC-dependent T cell priming as well as NOD1 signaling and inflammasome activity [please see (160) for review]. Our work suggests that sensitization to inhaled or subcutaneous antigens is defective in the absence of NLRP10 secondary to a DC migration defect; however T cell priming following topical sensitization was not evaluated (14). Therefore whether the NLRP10 and NLRP12 phenotype are complementary versus overlapping has yet to be determined. But interestingly both NLRP10 and NLRP12 were identified as susceptibility loci from GWAS studies of patients with atopic dermatitis (161, 162). NLRP10 is most highly expressed in the skin and knockdown of human NLRP10 in primary dermal fibroblasts attenuates innate immunity mediated by NOD1 (163). Therefore, further work is necessary to clarify if NLRP10 regulates adaptive immunity to skin antigens via hematopoietic cellular changes or rather acts in the barrier function of the skin.

## Category 3: Adaptive Immunity to Self Targets

Autoimmunity is a pathologic process wherein T and B lymphocytes are activated by self molecules and induce damage to host tissues. As tolerance is built into the education of T and B cells, an active process must overcome tolerance to induce lymphocyte sensitization to self molecules; the innate immune system plays one key role in that process. Much of the early work on NLRs and inflammasomes followed the discovery that constitutively active mutants of NLRP3 were responsible for a subset of periodic fever syndromes now grouped under the term autoinflammatory diseases [for review, see (164)]. This NLRP3-dependent collection of rare syndromes, called the Cryopyrin-associated periodic syndromes (CAPS), is characterized by self-limited bouts of inflammation of the joints and skin with more systemic inflammation depending on the subtype; all are accompanied by fever and elevated levels of IL-1β (165). In fact, these disorders are now successfully treated by blocking IL-1β activity (166). It is important to recognize that these disorders do not include self-reactive T or B cells and therefore are distinct from autoimmune diseases (167). Although both autoinflammatory and autoimmune diseases involve recurrent episodes of inflammation (sometime with similar cytokine profiles) triggered by self molecules, the cells that drive inflammation and the nature by which they do so in the two disease categories are different. Therefore for the remainder of this section, we will only consider the role of NLRs in the adaptive immune response to self targets and therefore autoimmune diseases. In contrast to the clear mechanistic link between autoinflammatory diseases and NLR activity, the link between autoimmunity and NLR activity is more tenuous (168).

How would NLR activity drive autoimmunity? One obvious link between inflammasome-forming NLRs and adaptive immunity is the potent cytokine IL-1β. IL-1β enhances expansion and survival of T cells (169), promotes differentiation of potentially pathogenic Th17 cells (170, 171) and can promote APC migration, thereby potentially enhancing antigen presentation (97) (Figure 2). Yet IL-1β cannot be sufficient for driving autoimmunity as autoreactive T and B cells are not a part of the pathophysiology of autoinflammatory diseases (165, 167). Indeed there is only limited evidence that NLR triggering induces a complete DC maturation program the way TLR activation transforms naïve DCs into potent APCs. Yet as discussed previously, particular NLRs such as CIITA, NLRC5, NLRP10, and NLRP12 regulate key steps in the antigen presenting function of DCs (34) and might regulate adaptive immunity to self in this way.

### Multiple sclerosis

Perhaps the strongest link between an inflammasome-forming NLR and an autoimmune process is the link between NLRP3 and multiple sclerosis (MS). MS is a Th1 and Th17-driven response directed against myelin-producing oligodendrocytes in the CNS (172). Overexpression of IL-1β, IL-18, and caspase-1 has long been recognized in samples from MS patients (173–175). Studies in animal models further strengthened a connection between inflammasome activity and MS. A murine model with a clinical phenotype similar to MS, experimental autoimmune encephalomyelitis (EAE) exists. However this model relies on a highly artificial induction step to induce CNS-reactive T cell priming using CFA administered with peptides from the CNS (14). As *Mycobacterium* can activate multiple NLRs including NOD1, NOD2, and NLRP3 (66, 80, 176), one might expect to observe differences in adaptive immune-mediated disease when the adjuvant contains *Mycobacterium* as does CFA. Despite bypassing the natural immunization process, this animal model has been useful to dissect the immune pathways governing the autoimmune destruction of the CNS. Indeed caspase-1-deficient mice have reduced disease severity and delayed onset of paralysis (177). Further, caspase-1 dependent IL-1β and IL-18 from activated DCs promotes Th17 and γδ T-cell activation directed against the CNS (178, 179).

Once the link between caspase-1 and NLRP3 was identified, an obvious question was whether this particular NLR regulates the adaptive immune response to the CNS. Yet the answer remains ambiguous. One group using two different models of EAE found that NLRP3-deficient mice had normal CD4^+^ T cell activation to CNS antigens but poor Th17 and Th1 differentiation. This was accompanied by reduced adaptive immune cell infiltration of the CNS and improved clinical outcomes (180, 181). Consistent with this finding, another group subsequently reported that NLRP3-sufficient DCs were required in order to program the primed autoreactive CD4^+^ T cells to traffic into the CNS (182). In contrast to the above work, a third group demonstrated, using a similar EAE model that NLRP3 deficiency did not affect the development of paralysis and presumably retained autoreactive T cell activity (183). Instead this latter study proposed an inflammasome-independent role for the adaptor ASC in the autoimmune disease process; however, subsequent work from this group demonstrated a second mutation in their strain of ASC-deficient mice in the guanine nucleotide-exchange factor Dock2, which regulates actin polymerization during lymphocyte migration and DC antigen uptake (184). Therefore, the findings from the 2010 paper might be affected by a non-inflammasome related mutation in the ASC-deficient mice used. Nevertheless, the findings in NLRP3-deficient mice should have been unaffected and therefore still suggest NLRP3-independent induction of adaptive immunity in EAE. To date, no clear mechanism explains the discrepancy in results between groups using NLRP3-deficient mice; however, a recent paper by Inoue et al. suggests that the dose of *Mycobacterium* used in the CFA adjuvant determines whether a NLRP3-dependent or -independent response is observed in EAE models (185). Since, in all of the papers discussed above the dose of *Mycobacterium* used was not specified, it remains unclear if a difference in mycobacterial doses explains the above discrepancy. Therefore the question remains whether an inflammasome-dependent step is required to initiate adaptive immunity to CNS self antigens.

We recently described another NLR crucial in the early stages of T cell priming against self-molecules in the CNS in a non-inflammasome-dependent manner. NLRP10 regulates the movement of antigen-laden DCs and in a standard EAE model, NLRP10 deficiency almost completely abrogated the priming of IL-17 and IFN-γ producing CD4^+^ T cells to CNS peptides and the associated demyelination (14). In this case, IL-1β and IL-18 production were not altered but rather autoreactive T cells were not activated because the crucial step of antigen presentation in secondary lymphoid organs failed. Again, given the use of CFA in this model, one can only conclude that DC movement and T cell priming are NLRP10-dependent in MS if the immune system is triggered in an analogous way during the natural course of disease.

### Diabetes

In contrast to MS/EAE, no other autoimmune disease has been as clearly linked to the activity of an NLR. Type 1 diabetes (T1D) is driven by T cells specific for beta cells in the pancreas ultimately resulting in destruction of the islets and is accompanied by systemic production of islet-specific antibodies. Although there is mounting evidence that NLRP3 inflammasome activity promotes insulin resistance in models of type 2 diabetes (T2D) (186–188), there is scarce evidence indicating that the same is true for autoimmune T1D. In fact, the unaltered development of spontaneous diabetes in NOD mice lacking caspase-1 argues against a role for inflammasome activity in the pathogenesis of autoimmune diabetes (189). Yet various human studies have suggested that particular polymorphisms in NLRP1 and NLRP3 confer risk for T1D (190, 191). Further, IL-1β is known to promote beta cell secretory dysfunction and apoptosis and this has prompted clinical trials of IL-1 blockade in both T1D and T2D. Unfortunately though, this approach was shown to be ineffective in new onset type 1 diabetics in a recent multicenter randomized double-blind placebo-controlled trial (192). Older work using the mouse NOD model suggested that IL-1R blockade in fact does not alter insulitis or the autoimmune process, although glycemic control was improved (193). Therefore, inflammasome activity and IL-1β in particular might play a more significant role in insulin sensitivity and beta cell dysfunction (T2D) rather than regulating autoimmunity to beta cells (T1D).

### Rheumatoid arthritis

The pathology in rheumatoid arthritis (RA) is driven by a T-cell-mediated immune response directed at the synovial lining of the joints and is accompanied by autoreactive antibodies systemically. Unlike some of the other autoimmune diseases discussed above, IL-1β plays a less significant role in the pathology of RA (194) and therefore the role of NLRs and inflammasomes in driving the autoimmune process has been less extensively studied. A handful of studies specifically looking for polymorphisms in NLRs or associated inflammasome components has indeed found some associations with NLRP1 and NLRP3 polymorphisms and RA susceptibility (195, 196). Yet two groups using two different mouse models of antigen-induced arthritis reported that the adaptive immune response directed at the joints was NLRP3, NLRC4, and caspase-1 independent (197, 198). Interestingly, both reported dependence instead on the inflammasome adaptor ASC. But as described above, one of these groups subsequently discovered an unintended mutation in a non-inflammasome pathway in the ASC-deficient mice relevant for antigen presentation by DCs and T cell activation (184). Given that both groups observed T cell and DC intrinsic defects in their strain of ASC-deficient mice, it is again possible that the above finding stems from an ASC-independent defect. Yet other data from these papers suggests that NLRP3, NLRC4, and caspase-1 do not appear to play a significant role in the adaptive immune response in RA models.

### Lupus

Systemic lupus erythematous (SLE or lupus) affects a wide range of organs primarily driven by immune complexes of nuclear antigens bound by autoantibodies, which initiate inflammation and tissue destruction. The primary nuclear antigen, double stranded DNA (dsDNA) can potentially act as a DAMP to trigger many PRRs including TLR7 and TLR9 (199), NLRP3 (200), and AIM2 (201) and each of these receptors has been implicated in the pathogenesis of lupus. In the case of the latter two PRRs, inflammasome function has been suggested from older studies demonstrating a crucial role for IL-1β (but not IL-1α) in the development of anti-nuclear antibodies and lupus disease manifestations (202). Indeed a recent elegant study by Shin et al. identified how NLRP3 is triggered by dsDNA; human monocytes produce IL-1β only when stimulated with dsDNA in combination with anti-dsDNA antibodies, the complexes known to trigger inflammation in lupus (200). Further, supernatants from these stimulated monocytes promoted IL-17 production from memory CD4^+^ T cells. This suggests that the complexes formed in lupus patients can activate myeloid cells via NLRP3 and thereby reinforce T cell activation. It remains unclear though if this process and the NLRP3 inflammasome are required for initiation of the self-directed adaptive immune process. In fact a recent limited genetic analysis of *Nlrp3* (and *Aim2*) failed to find an association of particular single nucleotide polymorphisms (SNPs) with SLE predisposition; instead, polymorphisms in *Nlrp1*, including one in the promoter region, were associated with SLE in a Brazilian population (203).

Interestingly, non-synonymous coding-region and promoter polymorphisms in *Nlrp1* have been recurrently identified in genetic screens for a number of autoimmune diseases including vitiligo (melanocytes in the skin and hair targeted), Addison’s disease (cortex of the adrenal gland targeted), type 1 diabetes (beta cells in the pancreas targeted), systemic sclerosis (nuclear antigens targeted), RA (synovium in joints targeted), and SLE (nuclear antigens targeted) (190, 191, 195, 203–206). Frustratingly, there has been little work done to identify how these various polymorphisms influence autoimmunity susceptibility outside of a possible effect on transcription level of *Nlrp1* (195). Indeed little is known about the function of NLRP1 *in vivo*, in part due to the divergence of *Nlrp1* genes between humans and mice. But of all NLRs discussed, NLRP1 is the most widely implicated in susceptibility to autoimmunity in human studies. Until a mechanistic understanding of these SNPs is discovered, the association with autoimmunity does not provide a paradigm in which to develop a role for NLRP1 in the initiation of adaptive immune processes directed against self molecules.

## Category 4: Adaptive Immunity to Altered Self Targets

As tumors are derived from host cells that have lost the ability to regulate their appropriate growth, there is little to mark them as foreign or dangerous to the immune system. Outside of the aberrant loss of particular inhibitory signals to immune cells or altered forms of a self molecule, one signal that might alert the innate immune system to the loss of homeostasis is excessive cell turnover and damage to surrounding tissue, leading to the release of DAMPs. Therefore NLRs might be a relevant pathway to detect unchecked proliferation in tumors and initiate an immune response to these altered host-derived cells. However, several studies have suggested that IL-1β may have a role instead in promoting tumorigenesis (207, 208), since reducing or eliminating IL-1β can prevent tumor metastases and progression (209, 210), thus suggesting that inflammasome signaling may play a more complex role in the tumor-immune system interaction.

### Colon

Most studies of NLR function in tumorigenesis have focused on colonic inflammation and tumor models. NLRs are involved in the maintenance of gut homeostasis and dysregulation of this fine-tuned balance can lead to chronic inflammation (please see Category 5: Adaptive Immunity to Foreign Self Targets), which is believed to create a tumor-promoting condition for intestinal cancer. Accordingly, mice deficient in caspase-1, ASC, NLRP3, NLRP6, NLRP12, and NLRC4 all have increased colitis and subsequent development of colorectal cancers in gut irritant models. In most of these studies caspase-1 processed IL-18 is a key regulator of these processes (211–214). Although most of these chronic models did not evaluate potential alterations in anti-tumor adaptive immunity, one study showed that in the absence of NLRP3 and caspase-1, reduced IL-18 in a colorectal tumor model led to diminished IFN-γ levels in the colon (214). However, the cellular origin of this IFN-γ was not identified and therefore it remains unclear if anti-tumor adaptive immunity was in fact altered. In contrast, IL-18 has been implicated in promoting tumor metastasis from the lung, but in a T-cell and B-cell independent fashion; tumor-derived IL-18 promoted increased expression of the inhibitory receptor PD-1 on NK cells and prevented effective immunosurveillance (215). Therefore there might be a very different effect of inflammasome activity outside of the gut, wherein secreted IL-1β and IL-18 actually suppress tumor surveillance by innate cells but perhaps ultimately promote adaptive immunity to tumor antigens.

### Tumor vaccines

Chemotherapy is one of the most commonly used treatments for cancer patients. It has been proposed that chemotherapy works via eliminating immunosuppressive cells and by directly inducing tumor-cell death (216, 217). Ghiringhelli et al. demonstrated that chemotherapy-treated dying tumor cells can activate the NLRP3 inflammasome in DCs and that a functional NLRP3 inflammasome in DCs is required for tumor-specific CD8+ T cell priming (217). In this study, the authors found that NLRP3- and caspase-1-deficient mice are unable to prime CD8^+^ T cells, unless exogenous IL-1β is provided. Furthermore, they found that HMGB1 from dying tumor cells is critical for IL-1β release by DCs. In contrast, van Deventer et al. found that NLRP3 activity actually impairs anti-tumor DC-based vaccination by enhancing the accumulation of tumor-associated myeloid-derived suppressor cells (MDSC) thereby inhibiting the cytotoxic T cell response (218). Tu et al. also found enhanced MDSCs in gastric tumors induced by overexpression of IL-1β in the stomach; however, the enhanced tumor development was independent of T and B lymphocyte responses (219). In line with these studies, Bruchard et al. demonstrated that although chemotherapy depleted MDSCs and increased the survival of tumor-bearing mice by generating tumor-specific CD8^+^ T cells, it triggered the release of cathepsin B from MDSCs leading to IL-1β secretion (220). IL-1β promoted IL-17 production from CD4^+^ T cells, which in turn attenuated the anti-tumor effect of chemotherapy via an IL-17-dependent proangiogenic effect (220). These conflicting results may be due to the ability of particular drugs to induce immunogenic tumor-cell death (221) or differences in the form of tumor antigens used in these studies, [i.e., particulate (217) versus soluble (218)], which might be taken up by different DC subsets or recognized differently by the innate immune system.

Although TLR ligands have been tried as adjuvants to enhance vaccination efficiency for many years, very limited work has been done with NLR ligands. In a recent study, Garaude et al. demonstrated that introducing the bacterial protein flagellin, which activates TLR5, NAIP5, and NLRC4, into tumor-cell lines induced a potent CD8^+^ T cell anti-tumor adaptive immune response and thereby helped eliminate injected tumors. They additionally found that priming of tumor-specific CD4^+^ and CD8^+^ T cells by DCs was promoted by the dual triggering of TLR5 and NLRC4 (222). Therefore, under specific situations, NLR triggering, possibly in combination with other PRRs, crucially regulates adaptive immunity to altered host cells in tumors.

## Category 5: Adaptive Immunity to Foreign Self Targets

Up to 100 trillion microbes inhabit the human intestinal tract, which is 10-fold the number of human cells in the body. This ecosystem consists of fungi, bacteria, archaea, and viruses; most of these organisms are not pathogenic, but rather commensal, meaning that both the host and the organism benefit from co-habitation. Since these organisms function as a part of the human body and the immune system tolerates their presence, they can be thought of as an immune target that is both foreign but also self. A majority of these microbes can be found in the colon, where they contribute to the energy we extract from food, defend the mucosa against invading pathogens, induce the production of protective mucus and antimicrobial peptides as well as influence host immunity (223, 224). The microbiota is needed to adequately develop gut-associated lymphoid tissues by recruiting IgA-producing plasma cells and CD4^+^ T cells to the lamina propria and directing the development both of local lymphocyte subsets (224–226) including Th17 and Treg populations, as well as distal B cell and T cell zones in lymph nodes and the spleen (227). Accordingly, changes in the microbiota are believed to contribute to the development of intestinal diseases as well as systemic metabolic disorders.

To date NLRs, which are clearly present and highly active in the gut, have not been implicated in educating these intestinal lymphocyte populations. However, their activity can have a profound impact on the composition of the microbiota and loss of NLRs with the ensuing dysbiosis can impact both local and systemic immunity (12, 227). The gut immune system must be able to tolerate commensal microbes, while still being able to keep microbes from coming in close proximity to the epithelial cell layer and inducing damage; the ability of NLRs to identify pathogens that have breached cell membranes makes them well suited to act at this level of barrier function (Figure 1). Several examples have been described: decreased NLRP3 expression and defective NOD2 have been associated with Crohn’s disease, in which the microbiota is believed to contribute to the intestinal inflammation (10, 228, 229); NLRP3 and NLRP6 deficiency lead to a high susceptibility to dextran sodium sulfate (DSS)-induced colitis (54, 230) and NLRP6 was shown to impact the composition of the microbiota leading to an intestinal dysbiosis that resulted in spontaneous intestinal inflammation (54). Huber et al. found an important role for NLRP3 and NLRP6 in regulating tissue repair and tumorigenesis in the colon through IL-18-dependent downregulation of dendritic cell-derived IL-22 binding protein (231). Conversely, inflammasome regulation of adaptive immunity at distal sites can be influenced by the gut microbiota. Ichinohe et al. demonstrated that commensal bacteria were critical for the induction of adaptive immune responses (including CD4^+^ T cell, CD8^+^ T cell, and antibody responses) to respiratory influenza infection by providing “signal 1” or the priming signal for inflammasome substrates pro-IL-1β and pro-IL-18 (232).

However, the impact of NLRs on the induction of adaptive immune responses in the intestine needs further elucidation. We have previously proposed a “two-hit model” of PRR triggering to set thresholds for adaptive immunity in which DCs are primed by TLR activation but licensed by NLR activation (34). This model could also work to maintain homeostasis in the gut, in which the presence of microorganisms could lead to activation of TLRs, but activation of NLRs by DAMPS would be the decisive step in initiating an adaptive immune response.

## Conclusion

Although a tremendous amount of work has been done resulting in significant advances regarding our understanding of NLR function over the past decade, there remains limited evidence that NLRs directly regulate adaptive immune responses. In ways distinct from TLR-driven pathways, NLRs indeed regulate aspects of DC migration, antigen presentation, and production of particular pro-inflammatory cytokines that can shape developing T cell responses (Figure 2). We have previously argued that this potentially represents a division of labor between two of the major subsets of PRRs, the TLRs and NLRs (34), although some NLRs can instigate inflammatory processes that overlap or modulate TLR-driven pathways to impact adaptive immunity. Yet for a majority of NLRs, including those identified through unbiased genetic screens such as NLRP1 (see Category 3: Adaptive Immunity to Self Targets), we do not know how these innate immune molecules function to instruct immunity. Given the location of these molecules and the distinct set of insults that stimulate their activity, it is not surprising that they have thus far been found to fulfill specialized but delimited functions in the intricate interplay between the innate and adaptive branches of the immune system.

## Conflict of Interest Statement

The authors declare that the research was conducted in the absence of any commercial or financial relationships that could be construed as a potential conflict of interest.
